# Changes to Euchromatin on LAT and ICP4 Following Reactivation Are More Prevalent in an Efficiently Reactivating Strain of HSV-1

**DOI:** 10.1371/journal.pone.0015416

**Published:** 2010-11-04

**Authors:** Clinton C. Creech, Donna M. Neumann

**Affiliations:** 1 Department of Ophthalmology (LSU Eye Center of Excellence), Louisiana State University Health Sciences Center, New Orleans, Louisiana, United States of America; 2 Department of Genetics, Louisiana State University Health Sciences Center, New Orleans, Louisiana, United States of America; University of Minnesota, United States of America

## Abstract

**Background:**

Epigenetic mechanisms, via post-translational histone modifications, have roles in the establishment and maintenance of latency of the HSV-1 genome in the sensory neurons. Considering that many post-translational histone marks are reversible in nature, epigenetic mechanisms may also play a critical role in the process of induced HSV-1 reactivation.

**Methodology/Principal Findings:**

This study utilized the rabbit ocular model of HSV-1 infection and reactivation, induced by the transcorneal iontophoresis of epinephrine (TCIE), to characterize changes to chromatin that occur between 0.5 and 4 h following the application of the reactivation stimulus. Our goal was to explore the hypothesis that chromatin remodeling is an early and essential step in the process of HSV-1 reactivation. Analysis of the HSV-1 latently infected rabbit trigeminal ganglia (TG) showed that enrichment of the euchromatic marker H3K4me2 significantly decreased in the LAT 5′exon region (∼2.5-fold) and significantly increased in the lytic ICP4 promoter region (∼3-fold) by 1 h post-TCIE in the highly efficient reactivating McKrae strain of HSV-1. In contrast, we observed no significant change in the euchromatic marks of H3K4me2 associated with LAT 5′exon or ICP4 promoter regions of the poorly reactivating KOS strain of HSV-1 following TCIE through 4 h. The implication that these observed epigenetic changes were linked to transcriptional activity was confirmed by qRT-PCR examining both LAT and lytic transcript abundance following TCIE. We found a significant decrease in the abundance of LAT RNA by 2 h post-iontophoresis of epinephrine coupled to an increase in the transcript abundance of ICP4 in the McKrae strain of HSV-1. By comparison, we observed no change in the LAT or ICP4 transcript abundance of the poor reactivator KOS following iontophoresis of epinephrine through 4 h.

**Conclusions/Significance:**

Our results implicate that chromatin remodeling is an early and essential step involved in the process of *in vivo* HSV-1 reactivation.

## Introduction

**Figure 1:**
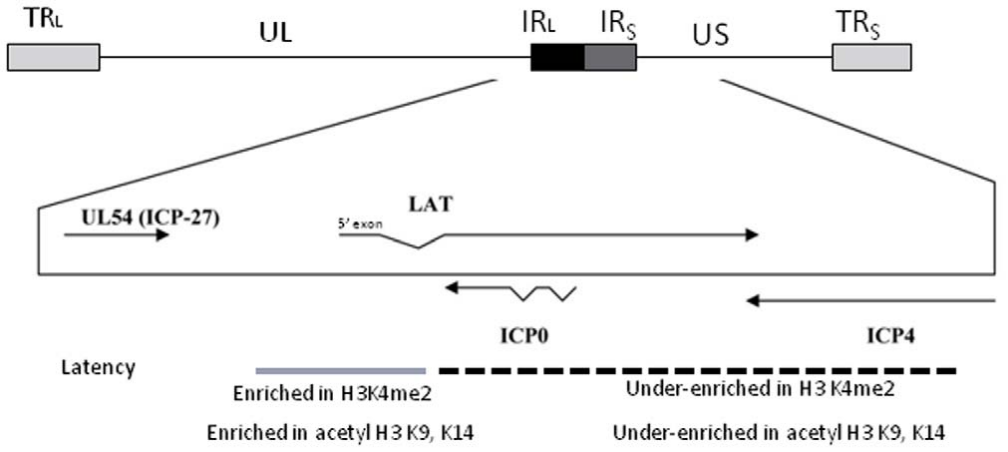
Diagram of the latent HSV-1 genome. Regions of long and short repeats (R_L_ and R_S_) have been analyzed for H3K4me2 and acetyl H3 K9,K14 enrichment [20], [21], [23]. During latency, the ICP0 and ICP4 regions of the genome are under-enriched in euchromatin, relative to the LAT 5′exon. This juxtaposition of euchromatin and heterochromatin allude to the likelihood of segregation of chromatin domains during latency.

Herpes simplex virus 1 (HSV-1) has the ability to establish a lifelong latent infection in the host [1]. During latency, the HSV-1 genome exists as a circular episome associated with histones [2]–[5], and only the latency-associated transcript (LAT) is abundantly transcribed [2]–[4]. The LAT region has been implicated in numerous viral functions, including the establishment of latency, suppression of latent transcription, and reactivation from latency [1]–[3], [6]–[12]. However, the exact mechanism of the LAT in reactivation of HSV-1 has yet to be elucidated. Recently, studies examining the viral genome during the primary HSV-1 infection *in vitro* have shown that H3 is associated with HSV-1 DNA in the initial stage of the infection, further suggesting that productive HSV-1 infections maintain covalent histone modifications that are typically representative of transcribed cellular genes [5], [13]–[17]. Subsequently, a number of key findings from studies designed to decipher LAT function have focused on potential epigenetic mechanisms involved in the establishment and maintenance of HSV-1 latency [18]–[23]. Critical examples include findings that show that during latency the LAT promoter and the LAT 5′exon (a gene region containing an enhancer element and critical for reactivation [20], [24]) regions are highly enriched in the transcriptionally permissive (euchromatic) histone marker acetyl H3 K9, K14 when compared to the immediate early (IE) promoters of ICP0, ICP4, and ICP27 in the footpad and ocular infection mouse models [19]–[22]. Furthermore, Wang *et al*.,[18] reported that lytic promoters become more associated with the repressive histone marker H3K9me2 and less associated with the euchromatic marker H3K4me2 in latently infected mouse ganglia. Taking into consideration the proximity and spatial arrangement of the IE promoters to the LAT, and specifically to the reactivation critical LAT 5′exon region of the HSV-1 genome (**Fig.1**), this compartmentalization of chromatin suggests that the latent HSV-1 genome may be organized into distinct chromatin domains that segregate euchromatic and heterochromatic regions of HSV-1 in order to maintain the integrity of the latent infection in the neuron [25]. Nonetheless, while it is becoming evident from these published reports that there are epigenetic components involved in the establishment and regulation of HSV-1 latency, the mechanisms by which HSV-1 reactivates from latency are still poorly defined, particularly in the animal model. Two recent independent reports confirmed that IE regions of latent HSV-1 are repressed, at least in part, through the deposition of facultative heterochromatin (indicated by triMeH3K27 enrichment) on the IE promoters and this deposition is regulated by LAT [26], [27] and an additional report has also proven that the inhibition of the histone demethylase LSD-1 using monoamine oxidase inhibitors results in the recruitment of repressive histone marks and has the capability of blocking HSV-1 lytic replication and reactivation from latency *in vitro* and in explanted ganglia [28]. These findings may be significant with respect to the overall mechanism of HSV-1 reactivation since facultative heterochromatin can interconvert between euchromatin (transcriptionally active) and heterochromatin (transcriptionally silent) via PTMs [29].

Previously, we showed that both the ICP0 and ICP4 promoter regions of HSV-1 become transiently enriched in the permissive histone mark acetyl H3 K9,K14 when sodium butyrate (NaB) is used as reactivation stimuli in mice latent with the efficiently reactivating 17*Syn*+strain of HSV-1 [19], [22], [30]. While NaB is known to have pleotropic effects *in vivo*, one fact that cannot be overlooked is that NaB is a known histone deacetylase inhibitor (HDACI), and as such, using NaB to stimulate *in vivo* reactivation in the mouse model has raised an important question with respect to the epigenetic regulation of HSV-1 reactivation; were the increased enrichments of acetylated histone marks on the IE promoters following NaB treatment merely a global consequence of the application of the HDACI and not a fundamental element in the molecular mechanisms that ultimately govern the process of HSV-1 reactivation? In order to answer this question and to begin to define the potential of epigenetic changes to the HSV-1 genome as a function of the reactivation process, we outlined studies with the following objectives: 1) to stimulate HSV-1 reactivation in an animal model using an efficient reactivation method not capable of directly modify histone tails; 2) to determine whether changes in the enrichment of a euchromatic histone marker could be observed for the LAT and IE regions of HSV-1 following reactivation stimuli; 3) to correlate any changes in the enrichments of euchromatic marks to LAT and lytic transcript abundance; and 4) to determine whether wild-type HSV-1 strains with different reactivation phenotypes established dissimilar profiles of euchromatic marker enrichments on the LAT and IE regions during latency and/or following the application of a reactivation stimulus. To accomplish these goals, we used the rabbit ocular model of HSV-1 infection and reactivation induced by the transcorneal iontophoresis of epinephrine (TCIE) to identify potential changes in the chromatin profiles of two phenotypically different wild-type HSV-1 strains. Strain McKrae is known for highly efficient reactivation following TCIE (the recovery of infectious virus in both the eye and TG occurs as early as 24 h post-treatment with 80–100% efficiency [31]–[33]), while strain KOS is typically known as a poor reactivator (reactivation efficiency for KOS is 5–20% following TCIE [34], [35]). While there have been reports showing that adrenergic agents can affect signaling pathways, thereby leading to enhanced gene expression,[36], [37] there is no evidence that epinephrine directly modifies histone tails and therefore made a logical alternative to HDACI as a reactivation stimulus in which to explore changes in chromatin patterns linked to the HSV-1 reactivation process. Finally, to perform these studies, we used the euchromatic histone marker H3K4me2 because it has been associated with both transcriptional memory and pol II activity [38], [39]. In summary, our results showed that enrichments of H3K4me2 on the LAT 5′exon and the IE region ICP4 rapidly and significantly changed post-TCIE in the McKrae strain of HSV-1. These changes in H3K4me2 enrichment correlated to significant decreased LAT RNA accumulation and a 2–6 fold increase in ICP4 transcript abundance following TCIE. On the other hand, when we explored the enrichment patterns of H3K4me2 following TCIE in the poor reactivator KOS [34], [35] we found no significant change in the H3K4me2 enrichments over time of these gene regions, nor could we discern any change in LAT or ICP4 transcript abundance through 4 h post-TCIE. Our results substantiate an epigenetic component is likely involved in the molecular mechanisms that potentially govern HSV-1 reactivation.

## Materials and Methods

### Ethics statement

The animal studies were approved by the Institutional Animal Care and Use Committee of the Louisiana State University Health Sciences Center, New Orleans (Institutional animal welfare assurance no. A3094-01 date of assurance 12/01/08.

Expires 12/31/12 and LSUHSC IACUC approval no. 2679- date of approval 6/03/2009 expires 6/01/2012). New Zealand White rabbits (1.5–2.5 kg) were used in all experiments and were handled and maintained in accordance with the tenets established by the Association for Research in Vision and Ophthalmology in its resolution on the care and use of animals in research.

### Viruses and cells

Low passage stocks of HSV-1 McKrae and KOS were obtained from master stocks prepared by David Bloom (UF, Gainesville, FL). The virus was grown on primary rabbit kidney cells using Eagle's minimal essential medium (Invitrogen-Life Technologies, Carlsbad, CA) supplemented with 10% fetal calf serum, 292 µg of L-glutamine/ml, and antibiotics (250 U of penicillin/ml and 250 µg streptomycin/ml). Viruses were plaque purified a minimum of three times and viral titers were obtained using CV-1 cells.

### Rabbit Infections

**Figure 2:**
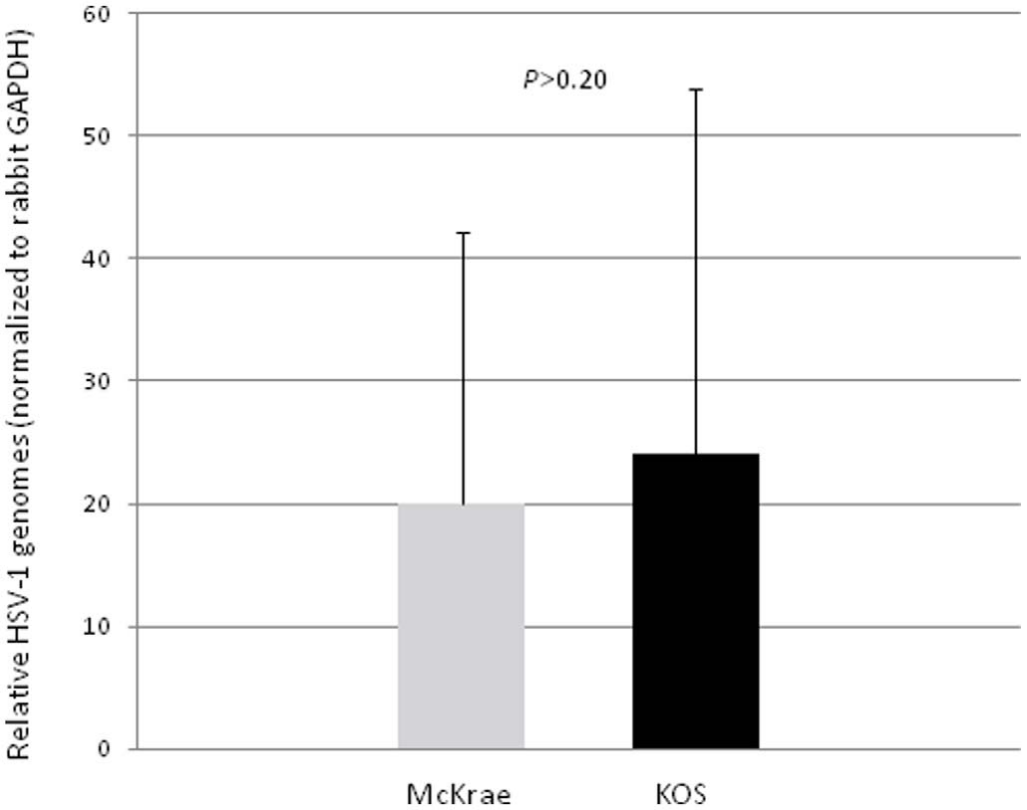
HSV-1 genome equivalents between HSV-1 strains McKrae and KOS. New Zealand White rabbits were inoculated in their corneas, following scarification, with viral titers of 200,000 pfu/eye for both McKrae and KOS strains of HSV-1. All infections were confirmed by slit-lamp examination. Equivalent latent infections between the two strains were confirmed by real-time PCR, measuring the relative quantity of HSV-1 genomes. All relative quantities of HSV-1 genomes were normalized to GAPDH quantities per sample (P>0.20). Relative quantities were calculated using a standard curve generated for each primer/probe set used.

Rabbits were anesthetized intramuscularly using a mixture of ketamine (30–45 mg/kg body weight) and xylazine (7.5 to 11.5 mg/kg body weight). Proparacaine drops were placed on the surface of the cornea to minimize pain during inoculation. A 2 by 2 crosshatch pattern was made on the surface of the corneal epithelium using a sterile 27-gauge needle and very light scratching. The viral inoculum (200,000 PFU/eye) was placed directly on the surface of the eye, and the lid of the eye was gently massaged to ensure even distribution of the virus. All infections were confirmed through slit-lamp examination (SLE) on post-inoculation (p.i.) day 3 using previously described methods [31]. Equivalent latent infections between the two strains was confirmed by real-time PCR, measuring the relative quantity of HSV-1 genomes present in the TG at >32 days p.i.(P>0.20) (**Fig. 2**).

### Rabbit Reactivation

Rabbits were considered latent at >28 days PI at which time no corneal or ocular lesions were detected by SLE. Rabbit corneas were subjected to TCIE using previously reported methods [31], [40]. Briefly, latently infected rabbits were anesthetized using ketamine/xylazine IM and subjected to transcorneal iontophoresis of epinephrine using a 0.015% epinephrine solution in water at 0.8 mA for 8 min per eye. Following the iontophoresis, rabbits were euthanized using an overdose of sodium pentobarbital at 0.5, 1, 2, 3, 4 or 8 h post-iontophoresis. The TG were rapidly removed and either processed for Chromatin ImmunoPrecipitation (ChIP) assays or placed in RNA*later* (Ambion, Inc., Austin, TX) for RNA extraction. Care was taken to ensure that all ganglia were processed post-mortem as quickly as possible (3–5 minutes per animal). Controls, latently infected rabbits, were not subjected to iontophoresis of epinephrine before ganglia extraction. All rabbits were subjected to ocular swabs peri-mortem to ensure that we could detect no spontaneous shedding of infectious virus prior to the onset of the experiment. Any rabbits with infectious virus in the ocular swabs were excluded from this study.

### ChIP assays

ChIP assays were performed as previously described [19], [20], [22], [23] with minor modifications made to the reagent volumes to accommodate rabbit TG. All solutions used before the collection of the chromatin antibody complexes contained protease inhibitors at the following concentrations: aprotinin (Sigma-Aldrich Corp., St. Louis, MO) 15 µg/ml; leupeptin (Sigma-Aldrich) 1 µg/ml; and phenylmethylsulfonyl fluoride (Sigma-Aldrich) 10 µg/ml. All steps were done at 4°C unless otherwise noted. The rabbit TG were rapidly removed from latently infected rabbits and individually homogenized in ice cold phosphate-buffered saline (PBS). Formaldehyde (final concentration of 1% [vol/vol]) was added to the homogenate to cross-link the chromatin. Samples were incubated at room temperature for 10 min on an orbital shaker with shaking. The cross-linking step was arrested using glycine (final concentration of 0.125 M) while the homogenate was again incubated at room temperature for 10 min on an orbital shaker with shaking. The homogenate was pelleted and washed three times with PBS containing the appropriate protease inhibitors. The homogenate was re-suspended in sodium dodecyl sulfate lysis buffer (Upstate, Millipore, Billerica, MA) and incubated on ice for 30 min. The cell lysate was sonicated to shear the chromatin into a population of fragments with an average size of 300–700 bp, as determined by agarose gel electrophoresis (1.6% gel). The sheared chromatin was diluted by the addition of 10 volumes of cold ChIP dilution Buffer (Upstate) and incubated with salmon sperm DNA-protein A-agarose (50% slurry) (Upstate) for 2 h to reduce the nonspecific binding. Beads were pelleted by centrifugation and the supernatant was decanted into a clean tube. A fraction representing 10% of the total sample volume was removed and placed on ice for further purification (see below). This fraction represents the input (IN) of the ChIP assay. The remainder of the supernatant was then incubated with anti-dimethyl histone H3 K4 (H3me2K4) (Upstate) at a concentration of 1 µl/ml overnight at 4°C with shaking. Chromatin-antibody complexes were collected by incubation with salmon sperm DNA-protein A agarose (50%) slurry and subsequent bead collection via centrifugation. The supernatants associated with the bead pellets were collected and stored at −80°C and represent the unbound (UB) fraction of the ChIP assay. The remaining bead pellets were then washed once in each low-salt, high-salt, and LiCl immune complex wash buffers, and then twice in Tris-EDTA buffer (all buffers, Upstate). Antibody-chromatin complexes were then eluted from the beads via incubation using freshly prepared elution buffer (0.1% SDS, 0.1 M sodium bicarbonate) heated to 65°C. These eluates represent the Bound (B) fractions of the ChIP assay. Both B and IN fractions were treated with NaCl (final concentration of 0.2 M) and were incubated at 65°C for 4 h. Samples were then treated with RNase A and proteinase K and the DNA was purified using a QiaQuick PCR purification kit (Qiagen).

### Real Time PCR analysis

Real time PCR was performed using TaqMan universal PCR master mix, No AmpEraseuracil N-glycolase (Applied Biosystems, Carlsbad, CA) and target specific primers and TaqMan dye labeled probe designed and generated by Applied Biosystems (Assays by Design part no. 4331348) in concentrations recommended by the supplier. Primer and probe sequences used for LAT 5′exon, ICP0, ICP4, GAPDH and rabbit centromere have been previously reported [20], [23]. All real time PCRs were performed and analyzed using a Biorad MyIQ Icycler. Cycle conditions used were as follows: 95°C for 10 min (1 cycle); and then 95°C for 15 sec, followed by 60°C for 1 min (45 cycles). Threshold values used for PCR analysis were set within the linear range of PCR target amplification.

### ChIP assay validation

All assays were validated using the cellular controls GAPDH (transcriptionally active) and rabbit centromere (transcriptionally silent), and only samples with >2-fold enrichment of H3K4me2 for GADPH were used in our final analyses.

### Determination of ChIP bound/input ratio

Relative enrichments of a given DNA region were determined by comparing the amount of the DNA region present in the post-IP fraction (bound) to the total DNA present in the sample (input), by expressing the ratio of bound/input (B/IN). All amounts are relative to a standard curve specific for the primer/probe set used. Standard curves were generated by using serial 10-fold dilutions of purified viral DNA or DNA purified from the rabbit TG. Each individual ChIP assay was analyzed as follows: First, triplicate PCR reactions were done for DNA purified from either bound or input fractions. Cycle thresholds (Ct) were then established within the linear range for each PCR and averaged. The average Ct for the B fraction and the average Ct for the IN fraction were then used to determine the relative quantity for the target DNA in each fraction by using the equation for the standard curve specific to the primer probe set used. The quantity was expressed as a ratio of the relative B quantity to the relative IN quantity (B/IN ratio). All samples were further normalized to the (B/IN) of the host gene, rabbit GAPDH.

### Analysis of rabbit TG for LAT, ICP0 and ICP4 RNA by qRT-PCR

Rabbit TG were isolated and placed in RNA*later* and stored according to the manufacturer's specifications. RNA was extracted by removing RNA*later* from samples and adding Trizol reagent (Sigma-Aldrich) to each sample. Briefly, each TG was homogenized in 1.2 ml Trizol and following the addition of 0.2 volume of chloroform, samples were centrifuged for phase separation. RNA was precipitated from the aqueous phase using 0.7 volume of isopropanol, followed by DNase treatment using DNA-*free* (Ambion), according to the manufacturer's directions. Reverse transcription using random primers was performed with High Capacity cDNA Reverse Transcription Kit (ABI), according to the manufacturer's instructions. Briefly, 20 µl reactions contained DNAse treated RNA, manufacturer supplied buffer, deoxynucleoside triphosphate mix, 1 µM random hexamer primer, 1 U RNase inhibitor (Ambion), and Multiscribe reverse transcriptase. In the case of ICP0, a 10 uL aliquot of purified RNA was used with the strand specific primer for the ICP0 transcript (LAT I-1, GACACGGATTGGCTGGTGTAGTGGG; nucleotides 120797 to 120820) [22]. All reaction conditions followed the manufacturer's instructions. Real time PCR reactions were performed on cDNA according to the above described procedures and protocols.

### Statistical analysis

Six to seven individual samples were used for ChIP assay at each individual time point and eight to ten individual samples were used in all RNA analysis. One Way Analysis of Variance (*ANOVA*) was used to determine the statistical significance of all data obtained and was performed by analyzing variables (B/IN ratios for each region normalized to GAPDH relative ratios) following TCIE, relative to the constant (latent) time point. All analyses were done using the statistical analysis software, StatPlus Professional version 2009, for Windows XP (AnalystSoft).

## Results

### The reactivation critical LAT 5′exon region of HSV-1 maintains a significantly enriched H3 K4me2 chromatin profile relative to the IE regions during latency in the rabbit TG

**Figure 3:**
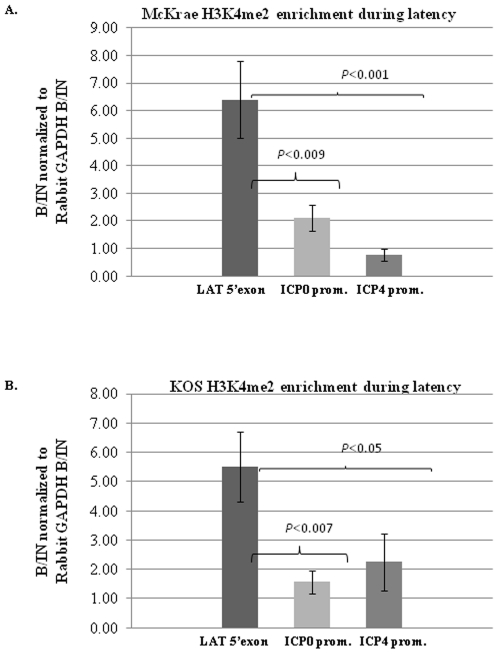
H3K4me2 enrichment during latency. **A.**) **H3K4me2 status of rabbit TG latently infected with wild type McKrae (n = 7).** **B.**) **H3K4me2 status of rabbit TG latently infected with wild type KOS (n = 7).** Rabbits were infected by corneal scarification with 200,000 pfu/eye of virus. ChIP analysis were performed as previously described [20], [21], [23] with TaqMan real-time PCR used for the analysis of the LAT 5′exon, ICP0 promoter and ICP4 promoter. B/IN ratios for each target gene were normalized to the B/IN ratios of rabbit GAPDH (cellular control). Real time PCR was performed with TaqMan universal PCR master mix, No AmpErase uracil N-glycolase and target specific primers and a fluorescently labeled probe (see references within). All PCR reactions were performed in triplicate and the average C(t) values were used to determine the relative quantity of DNA in either the bound or input fractions by using the equation for a standard curve specific to the primer/probe set used. The mean values are represented by the graph and the ±SEM is presented for each region.

We previously established that rabbits latent with the efficiently reactivating HSV-1 strain 17*Syn*+ were significantly enriched in H3K4me2 on the LAT 5′exon, but not on the ICP0 or ICP27 promoter regions [41]. However, in our previous experiences using TCIE as a reactivation stimulus in the rabbit, we obtain higher and more consistent episodes of HSV-1 reactivation, quantified by the presence of infectious virus in ocular swabs and TG, in the McKrae strain of HSV-1 rather than 17*Syn*+ [35], [42]. Therefore, we opted to use McKrae as our highly efficient reactivator for all experiments described in the scope of this study to limit the possible variability caused by less efficient reactivation in latent rabbits. To date, we could find no reports that established the latent chromatin profile of the HSV-1 strains McKrae or KOS in rabbits, specifically regarding the H3K4me2 enrichments of the LAT and IE regions of either viral strain. We performed ChIP analysis using the euchromatic H3K4me2 in rabbits latently infected with McKrae. We found that the LAT 5′exon region of the McKrae strain was significantly more enriched in H3K4me2 compared to the ICP0 promoter (P<0.009) as well as the ICP4 promoter (P<0.001) regions of the genome. In addition, we alse performed ChIP assays to assess the H3K4me2 enrichment of these regions in the KOS strain of HSV-1. We found a similar pattern of H3K4me2 enrichment in the KOS strain, where the LAT5′exon region of KOS was significantly enriched in H3K4me2 relative to the ICP0 promoter (P<0.007) and the ICP4 promoter (P<0.05) (**Fig.3**) (**Table 1**).

**Table 1:** H3K4me2 enrichment values during latency in the McKrae and KOS infected rabbit TG.

Virus, Target, expt. no	Normalized relative enrichment	Mean of normalized relative enrichment ±SEM^a^
**KOS**		
**LAT 5′ Exon**		
1	**1.78**	**5.52±1.26**
2	**1.14**	
3	**5.84**	
4	**9.69**	
5	**6.97**	
6	**5.6**	
7	**7.59**	
**ICP0 promoter**		
1	**2.45**	**1.56±0.39**
2	**0.981**	
3	**0.481**	
4	**1.49**	
5	**2.1**	
6	**2.92**	
7	**0.525**	
** ICP4 promoter**		
1	**1.85**	**2.25±0.96**
2	**1.27**	
3	**5.04**	
4	**0.131**	
5	**0.988**	
6	**6.08**	
7	**0.362**	
**McKrae**		
** LAT 5′ Exon**		
1	**6.2**	**6.4±1.41**
2	**4.34**	
3	**1.72**	
4	**8.1**	
5	**7.7**	
6	**4.13**	
7	**12.4**	
**ICP0 promoter**		
1	**3.66**	**2.1±0.46**
2	**2.77**	
3	**0.11**	
4	**2.13**	
5	**1.34**	
6	**2.47**	
7	**2.27**	
** ICP4 promoter**		
1	**0.49**	**0.77±0.22**
2	**0.68**	
3	**0.02**	
4	**1.53**	
5	**1.47**	
6	**0.74**	
7	**0.5**	

*^a^*Relative quantities were normalized to GAPDH as a cellular control.

### The H3 K4me2 enrichment and RNA abundance of the LAT 5′exon region significantly decreased at early time points after TCIE in McKrae strain

**Figure 4:**
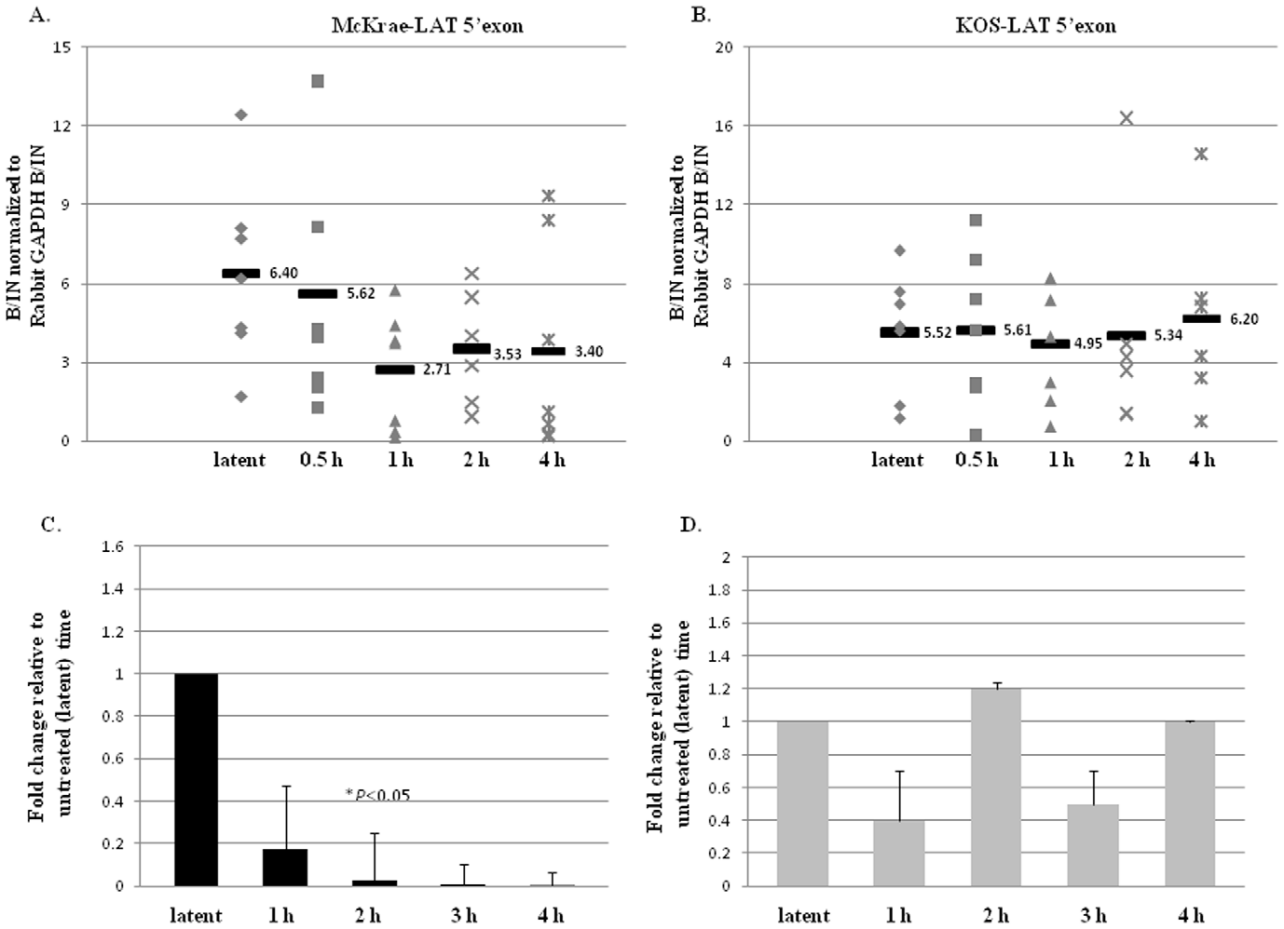
Changes in H3K4me2 enrichment and RNA abundance of the LAT 5′exon following transcorneal iontophoresis of epinephrine as a reactivation stressor in rabbits. All samples were validated using a transcriptionally active (GAPDH) and a transcriptionally repressed (centromere) gene. Samples were precipitated with anti-H3K4me2 and analyzed by real-time PCR. B/IN ratios for each target gene were normalized to the B/IN ratios of rabbit GAPDH (cellular control). The mean values are represented by a horizontal bar. All assays were further validated to ensure equivalent HSV-1 genome copies were present using GADPH. One TG per experiment was used (*n* = 6–7) for all ChIP assays. LAT 5′exon (X14112.1-nucleotides 119326–119397) **A.**) **H3K4me2 enrichment of the LAT 5′exon post-TCIE for the highly efficient reactivator McKrae:** ChIP analyses were performed post-TCIE at 0.5, 1, 2 or 4 h (indicated on X-axis). **B.**) **H3K4me2 enrichment of the LAT 5′exon post-TCIE for the poor reactivator KOS:** ChIP analyses were performed post-TCIE at 0.5, 1, 2 or 4 h. **C.**) **Relative change in LAT RNA of rabbits latent with McKrae following TCIE:** RNA was isolated using TRIzol reagent according to the manufacturer specifications. One rabbit TG was used per sample, and 8–10 samples were used for each time point. RNA was transformed to cDNA, and analyzed by real time PCR in triplicate. Relative quantities of were normalized to rabbit GAPDH. (There is no significant change in GAPDH expression following iontophoresis in the rabbit P>0.10) The error bars represent the positive standard deviation from the mean. The graphs are depicted as fold change in the RNA relative to the 0 h time, where the 0 h time was set to equal a value of 1. **D.**) **Relative change in LAT RNA of rabbits latent with KOS following TCIE.**

We previously showed in mice latent with HSV-1 strain 17*Syn*+ that upon the application of the HDACI sodium butyrate (used as an *in vivo* reactivation stimulus) [30], the LAT 5′exon region undergoes rapid deacetylation [19], [22] yielding a significantly less transcriptionally permissive chromatin profile. To determine whether chromatin remodeling of the LAT region is a consistent event potentially associated with HSV-1 reactivation, regardless of the reactivation stimulus applied, we assessed the H3K4me2 enrichment of the LAT 5′exon region of both the McKrae and KOS strains at 0.5, 1, 2, and 4 h post-TCIE. Latently infected rabbits were subjected to TCIE and the H3K4me2 enrichment of the reactivation critical LAT 5′exon region was measured via ChIP at latency, 0.5, 1, 2, and 4 h post-TCIE. Beginning as early as 0.5 h post-TCIE, we found a decrease in the H3K4me2 enrichment associated with the LAT 5′exon, with a significant (P<0.05) 2.5-fold reduction in the overall H3K4me2enrichment of LAT 5′exon by 1 h post-TCIE, relative to latency in the highly efficient reactivator McKrae (**Fig.4A**). Further, the LAT 5′exon region of McKrae strain remained under-enriched in H3K4me2 through 4 h post-TCIE relative to the latent time point. To ensure that the observed reduction was a consequence of chromatin remodeling and not due to an overall reduction in HSV-1 genomes present in the ganglia post-TCIE, all ChIP assays were validated by real-time PCR for HSV-1 genome quantity. Relative quantities of HSV-1 DNA pol were determined from each ChIP input fraction and normalized to GADPH [23]. One Way ANOVA indicated no significant difference in the number of HSV-1 genomes present in any of the samples (P>0.10). Therefore, our results show that the LAT 5′exon (a region critical for efficient HSV-1 reactivation) loses enrichment of the transcriptionally active histone mark H3K4me2 following the application of a reactivation stimulus in the efficiently reactivating McKrae strain. In contrast, when the same experimental protocol was applied to rabbits latent with HSV-1 strain KOS, with the goal being to determine whether we could observe a difference in the patterns of enrichment of H3K4me2 associated with the LAT region of KOS following TCIE of latently infected rabbits, we found no significant change (either increase or decrease) in the overall enrichment of H3K4me2 for the LAT 5′exon at any time through 4 h post-TCIE in HSV-1 strain KOS (**Fig.4B**).

We then examined whether the observed epigenetic changes could be correlated to changes in transcript abundance. We measured the RNA abundance of the LAT region during latency and at 1, 2, 3, and 4 h post-TCIE using qRT-PCR for both viral strains. We detected a∼5-fold decrease (P<0.09) in the LAT RNA accumulation by 1 h post-TCIE, and a significant decrease (∼10-fold) in the accumulation of LAT RNA by 2 h post-TCIE (P<0.05) compared to the latent values in the rabbits latent with the efficient reactivator McKrae. This decrease in RNA abundance of LAT was consistent through 4 h post-TCIE (**Fig.4C**). On the other hand, examination of the RNA accumulation by qRT-PCR following TCIE in rabbits latently infected with HSV-1 strain KOS showed no significant change in the accumulation of LAT RNA through 4 h post-TCIE when compared to latent values in the KOS strain of HSV-1 (**Fig. 4D**). These findings confirm that a correlation exists between the loss of euchromatic histone mark enrichment and the subsequent decrease in LAT RNA accumulation at very early times following the application of a reactivation stimulus in the rabbit model. The fact that the changes in H3K4me2 enrichments appear to precede the decreased LAT RNA accumulation coupled with the finding that this phenomenon can only be found in the highly efficient reactivator, McKrae but not in the poorly reactivating KOS strain at early times following the application of a reactivation stimulus present evidence that epigenetic mechanisms could likely be involved at early times in the HSV-1 reactivation process.

### The H3K4me2 enrichment of the ICP0 promoter does not significantly change post- TCIE

**Figure 5:**
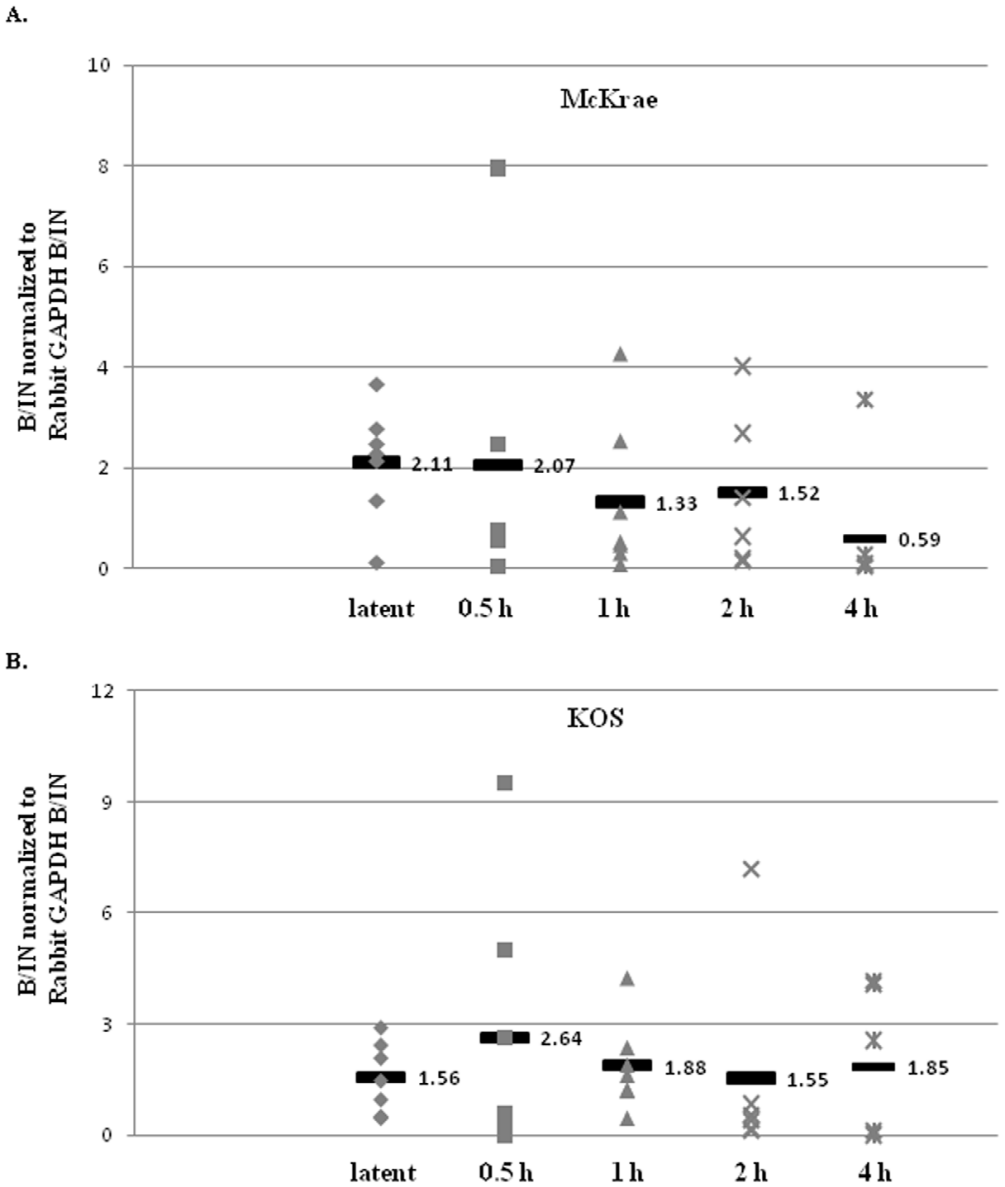
Changes in H3K4me2 enrichment of the ICP0 promoter following transcorneal iontophoresis of epinephrine as a reactivation stressor in rabbits. ChIP analyses were performed post-TCIE at 0.5, 1, 2 or 4 h (indicated on X-axis). **A.**) **H3K4me2 enrichment of the ICP0 promoter post-TCIE for McKrae Strain:** **B.**) **H3K4me2 enrichment of the ICP0 promoter post-TCIE for KOS strain:** Through 4 h post-TCIE, we observed no significant change in the H3K4me2 enrichment of the ICP0 promoter following TCIE in either McKrae or KOS strains.

To further explore the possibility that changes in H3K4me2 marks may play a role in reactivation by potentially activating lytic genes for transcription via PTMs, we analyzed the H3K4me2 enrichment of the ICP0 promoter post-TCIE in both McKrae and KOS strains at 0.5, 1, 2 and 4 h post-TCIE. ChIP analysis showed that the H3K4me2 enrichment of the ICP0 promoter did not change significantly in either strain through 4 h post-TCIE, relative to latency (P>0.11 –McKrae and P>0.78-KOS) (**Fig. 5A and B**). This finding was not anticipated, at least with respect to the McKrae strain, because previous studies using both *ex vivo* and *in vivo* reactivation stimuli in mice showed that the ICP0 promoter region of the 17*Syn*+ genome underwent transient H3 acetylation at early times after the reactivation stimuli were administered [19], [22]. Considering the similarities in the reactivation efficiencies and the latent chromatin profiles between the 17*Syn*+ and McKrae strains, we expected to find a similar pattern of chromatin remodeling in the latent rabbit TG subjected to TCIE. However, observed changes associated with ICP0 in the mouse model were transient and so the possibility remains that H3K4me2 enrichments of ICP0 may also be transient, occurring prior to the earliest assay time point. Nonetheless, the fact that H3K4me2 enrichment of the ICP0 promoter does not change following TCIE does indicate that the changes in H3K4me2 enrichment that are observed following TCIE in the LAT region of HSV-1 strain McKrae are likely not due to global changes in chromatin following epinephrine stimulation.

### The accumulation of ICP0 transcripts does not significantly change at early times post-TCIE in rabbit TG latent

We hypothesized that changes in the chromatin profiles associated with key regions of the latent HSV-1 genome might precede increased lytic transcript abundance. Still considering that transient changes in H3K4me2 marks associated with the IE lytic promoter ICP0 may have been missed due to experimental constraints, we assessed whether we could detect changes in the accumulation of ICP0 transcript abundance at early times post-TCIE. Unfortunately, we could detect no changes in the transcription of the ICP0 region by qRT-PCR following TCIE in either McKrae or KOS infected animals. While these results were disappointing, the inability to detect an increase in ICP0 transcript abundance by qRT-PCR at very early times following a reactivation stimulus has been reported in the literature, and therefore was not entirely unexpected [22]. The implications of this are further explored in the discussion section of this manuscript.

### Increased H3K4me2 enrichments of the ICP4 promoter occurs parallel to the decrease of H3K4me2 associated with the LAT 5′exon post-TCIE in McKrae

**Figure 6:**
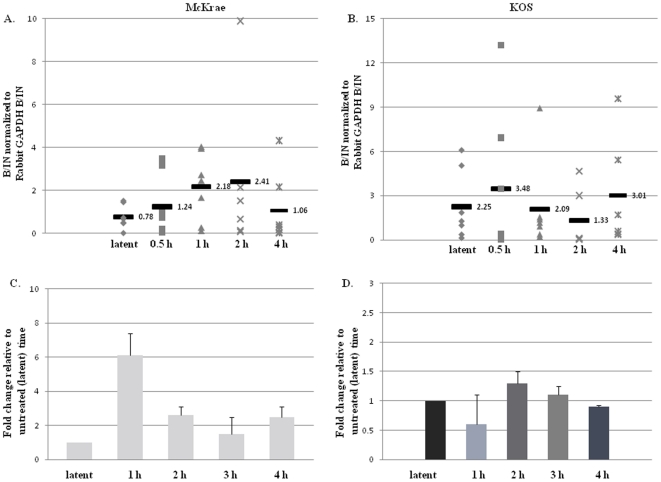
Changes in H3K4me2 enrichment and RNA abundance of the ICP4 promoter following transcorneal iontophoresis of epinephrine as a reactivation stressor in rabbits. All samples were validated using a transcriptionally active (GAPDH) and a transcriptionally repressed (centromere) gene. Samples were precipitated with anti-H3K4me2 and analyzed by real-time PCR. B/IN ratios for each target gene were normalized to the B/IN ratios of rabbit GAPDH (cellular control). The mean values are represented by a horizontal bar. All assays were further validated to ensure equivalent HSV-1 genome copies were present using GADPH. One TG per experiment was used (*n* = 6–7) for all ChIP assays. LAT 5′exon (X14112.1-nucleotides 119326–119397) **A.**) **H3K4me2 enrichment of the ICP4 promoter post-TCIE for the highly efficient reactivator McKrae:** ChIP analyses were performed post-TCIE at 0.5, 1, 2 or 4 h (indicated on X-axis). **B.**) **H3K4me2 enrichment of the ICP4 promoter post-TCIE for the poor reactivator KOS:** ChIP analyses were performed post-TCIE at 0.5, 1, 2 or 4 h. **C.**) **Relative change in ICP4 transcript abundance in rabbits latent with McKrae following TCIE:** RNA was isolated using TRIzol reagent according to the manufacturer specifications. One rabbit TG was used per sample, and 8–10 samples were used for each time point. RNA was transformed to cDNA, and analyzed by real time PCR in triplicate. Relative quantities of were normalized to rabbit GAPDH. (There is no significant change in GAPDH expression following iontophoresis in the rabbit P>0.10) The error bars represent the positive standard deviation from the mean. The graphs are depicted as fold change in the RNA relative to the 0 h time, where the 0 h time was set to equal a value of 1. **D.**) **Relative change in ICP4 transcript abundance in rabbits latent with KOS following TCIE.**

Subsequent ChIP analyses of the HSV-1 genome of the McKrae virus in the rabbit TG revealed that the H3 K4me2 enrichment of the ICP4 promoter significantly increased ∼3-fold by 1 h post-TCIE when compared to latency (P<0.04) (**Fig.6A**). Furthermore, we were able to detect increased H3K4me2 enrichment of the ICP4 promoter as early as 0.5 h post-TCIE, which remained consistent through 2 h post-TCIE. The increased H3K4me2 enrichment associated with the ICP4 promoter corresponds to decreased enrichment of the H3K4me2 of the LAT 5′exon in a parallel time frame. By 4 h post-TCIE our ChIP analyses showed that H3K4me2 enrichment of the ICP4 promoter region returned to latent enrichment profile again demonstrating that increased euchromatic histone mark enrichments on the ICP4 promoter region of HSV-1 are transient and occur rapidly following the application of a reactivation stimulus. The increased H3K4me2 enrichment indicates that the ICP4 promoter of HSV-1 undergoes chromatin remodeling to a more permissive chromatin profile, rapidly following TCIE. However, by comparison, when the same experimental parameters were applied to rabbits latently infected with the poor reactivator, KOS, we found no significant change in the overall H3K4me2 enrichment of the ICP4 promoter relative to the latent time point (**Fig. 6B**).

### ICP4 mRNA increases by 1 h post-TCIE in McKrae strain

To determine whether the increased enrichment of H3 K4me2 associated with the ICP4 promoter region of HSV-1 also correlated to an increase in transcript abundance of this region post-TCIE, we analyzed the mRNA abundance of ICP4 during latency as well as at 1, 2, 3, and 4 h post-TCIE in both McKrae and KOS strains using qRT-PCR Following TCIE, we were able to detect changes in the ICP4 transcript accumulation, and depending on the time point assayed, we found between an average 2–6 fold increase in ICP4 transcript abundance (P<0.07) (**Fig. 6C**). These findings are consistent with the observed changes in the chromatin profile of the ICP4 promoter region of the HSV-1 genome, which show that the chromatin associated with this region becomes more transcriptionally permissive with changes in chromatin noted as early as 0.5 h post-reactivation stimulus. In contrast, no appreciable increase or decrease in the transcript abundance of the ICP4 region of the poor reactivator, KOS, could be detected through 4 h post-TCIE (**Fig. 6D**). Again, these results remain consistent with the chromatin profile of the ICP4 promoter region of the KOS strain, showing that the chromatin associated with this region of the genome does not significantly change following the application of TCIE. Considering the kinetics of the display of chromatin modifications and subsequent changes to mRNA levels, this data also suggests that the transient enrichments of the transcriptionally active histone mark, H3K4me2, precede an increase in ICP4 transcript accumulation following TCIE.

## Discussion

It has been hypothesized that specific patterns of post-translational histone tail modifications serve as critical markers with the capability of dictating the transcriptional accessibility of regulatory regions within the latent HSV-1 genome [20]. Previous studies showed that both the LAT promoter and LAT 5′exon (reactivation critical region containing an enhancer element) of the HSV-1 genome displayed a chromatin profile consistent with transcriptional permissiveness during latency, while the IE regions maintained a chromatin profile consistent with transcriptional repression [18]–[20]. Subsequent studies identified key changes in the acetyl H3 K9, K14 associated with the LAT and IE regions of HSV-1 upon the application of an HDACI as a reactivation stimulus in the mouse model [19]. However, it must be noted that H3 acetylation may not be an accurate predictor of gene expression after treatment with an HDACI [43] and these previous reports failed to connect epigenetic changes to the latent viral genome with increased lytic transcription, raising the question as to the relevance of epigenetic changes in reactivation.

The H3K4me2 marker is a dynamic and reversible epigenetic marker associated with active transcription [38], [44]. That, coupled with the reversibility of the latent stage of the HSV-1 life cycle and the recent finding that facultative heterochromatin is deposited on the IE regions of HSV-1 during latency, adds credence that HSV-1 reactivation may be regulated epigenetically. Considering the implications of the aforementioned, the focus of this current study was to use ChIP assays profiling the H3K4me2 enrichments of the LAT and IE regions of the efficiently reactivating McKrae strain of HSV-1 with the transcriptional status of both LAT and lytic regions of HSV-1 following the application of a reactivation stimulus to determine whether epigenetic changes take place and to correlate any changes to increased lytic transcription. Our goal was to provide evidence that epigenetics could play a key regulatory role in the transition from latency to reactivation via histone tail modifications specific to active transcription. The implications of our findings are discussed below.

During latency the LAT 5′exon of the highly efficient HSV-1 reactivating strain McKrae is enriched in the post-translational histone modification H3K4me2. The H3K4me2 enrichment does not extend to the IE regions of ICP0 and ICP4 in the rabbit TG, a finding that remains consistent with the previously published reports. It has been suggested that the LAT 5′exon contains *cis* acting DNA elements that are responsible for the recruitment of histone modifying enzymes, such as methyltransferases, that help to establish a transcriptionally permissive environment [20]. Our data shows that the enrichment of euchromatin associated with the LAT 5′exon rapidly and significantly decreases in response to an external reactivation stimulus (relative to latency). However, when the same reactivation stimulus was applied to a poorly reactivating HSV-1 phenotype, KOS strain, we detected no significant change in H3K4me2 enrichment through 4 h post-TCIE. The fact that we can observe these epigenetic changes in the reactivation critical LAT 5′exon region of the highly efficient reactivator McKrae, but not in the poor reactivator, KOS clearly suggests that the chromatin remodeling that occurs in response to a reactivation stressor is a fundamental property associated with reactivation of HSV-1 rather than a consequence due to the nature of the stimulus or as a global response to epinephrine stimulation. We further extended our analyses to determine if the decrease in H3K4me2 enrichment associated with the LAT 5′exon could be correlated to a decrease in LAT transcript abundance subsequent to TCIE. Again, we showed a corresponding decrease in the transcript abundance of the LAT following iontophoresis in the efficient reactivator, McKrae, while rabbits latent with the poorly reactivating virus KOS had no significant reduction in either the H3K4me2 enrichment or in the LAT transcript abundance through 4 h post-TCIE. These data present evidence that the epigenetic changes observed in McKrae occur in response to the reactivation stimulus and are likely involved in early reactivation within the neuron.

Our data shows that epigenetic changes to chromatin associated with the LAT 5′exon occurs in parallel to the chromatin remodeling of the ICP4 promoter in the McKrae strain only. We show that the ICP4 promoter region of McKrae transitions to a more transcriptionally permissive state, relative to latency, very early after TCIE, and this increase in the euchromatic histone marker H3K4me2 can be correlated to increased ICP4 transcript abundance at very early times following stimulus application. Conversely, there is no significant change in the ICP4 H3K4me2 enrichment or the transcript abundance in the poorly reactivating strain of KOS.

Notably, we did not observe a significant increase in H3K4me2 enrichment associated with the ICP0 promoter region of HSV-1 after the application of our reactivation stimulus through 4 h post-treatment. This finding was unexpected, considering that it is accepted in the lytic infection that IE genes are activated in a cascade fashion, in which ICP0 is the first gene activated within that paradigm. Considering this, one would expect that the H3K4me2 enrichment profile of the ICP0 region would also change to a more transcriptionally permissive one in response to reactivation stimulus at early times. In fact, it has already been shown that the ICP0 promoter remodels to an acetylated state both *ex vivo* (explant-induced reactivation) and *in vivo* (HDACI induced reactivation) [19], [22]. Nonetheless, there are reports that instigate that H3 acetylation is not always an accurate predictor of gene expression in cases where and HDACI was used to stimulate activation of gene regions. Therefore, the disparity in our observations could be explained by at least two possibilities. The first is that H3 K4me2 associated with ICP0 undergoes remodeling in a rapid and transient manner, similar to what was observed for ICP4 post-TCIE in rabbit TG latent with McKrae. In such a scenario, one can envision that significant changes to chromatin associated with ICP0 can occur prior to our earliest assay time point of 0.5 h. We hypothesize that if remodeling of the ICP0 promoter does corresponds to the decrease in H3K4me2 associated with the LAT 5′exon, then ChIP analysis of earlier times post-reactivation stimulus may show an increase in H3 K4me2 associated with ICP0. Unfortunately, the time limitation of applying this method requires a minimum of 0.5 h and is a limitation of the experimental design. However, considering that we observed a significant 3-fold increase in H3K4me2 enrichment of ICP4 at 1 h post-iontophoresis, a second possibility must also be considered. The possibility remains that no enrichment of H3K4me2 associated with the ICP0 promoter occurs post-TCIE in McKrae. It has been widely reported that ICP0 is necessary for efficient reactivation from latency, [45]–[51] however; there are several studies that have indicated that, while important in the efficient reactivation from latency, the ICP0 protein is not required for the initiation of reactivation in quiescent cells or the latent neuron [52]–[55]. If one considers that ICP0 is not a necessary component in the initiation of reactivation from a latent neuron, combined with the hypothesis that epigenetics play a key role in the HSV-1 reactivation process it can also be expected that there will be a lack of measurable changes to the euchromatic markers associated with the ICP0 region of HSV-1 following TCIE. Nonetheless, it has been previously reported that ICP0 has chromatin modifying properties [56], [57] and has been implicated in the enrichment of acetylated histones at viral promoters [58]. Considering this, the expression of ICP0 could, in part, promote changes in the chromatin profiles of IE lytic promoters from a less transcriptionally permissive state to a more transcriptionally permissive one, [59] potentially causing a cascade of IE gene expression. Work aimed at confirming or refuting this hypothesis is currently underway.

Our results suggest that the LAT 5′exon remodeling from a transcriptionally active state to a less permissive one may facilitate lytic remodeling. Several recent studies identified regions within the HSV-1 genome that possess key elements consistent with cellular chromatin insulators [20], [59]. These insulator-like regions identified within the latent episome displayed characteristic enhancer blocking and silencing activities and give a plausible explanation as to why the LAT region (and specifically, the LAT 5′exon, containing and enhancer element) of HSV-1 is highly enriched in transcriptionally permissive histone modifications, while the adjacent ICP0 region remains under-enriched during latency [59]. These previous studies strongly indicate that preservation of unique transcriptional domains plays a key role in the maintenance of HSV-1 latency [14], [19], [20], [59]. Considering this, one could hypothesize that the disruption of this domain segregation via chromatin remodeling could result in HSV-1 reactivation from a latent state, and if chromatin insulators are responsible for the maintenance of the HSV-1 latent phase, then either a collapse or an alteration in histone modifying complex composition of such a key regulatory component could be a key step in the early events surrounding HSV-1 reactivation. We hypothesize that the LAT 5′exon is a major regulatory factor in the reactivation process, that recruits dynamic chromatin modifying enzymes that can, upon the initiation of reactivation, begin the process of establishing a transcriptionally unfavorable environment around the LAT by “collapsing” the integrity of this insulator-like region closest to this element within the context of the genome. Our data provide evidence that the transient increases in H3 K4me2 enrichment of the ICP4 promoter are linked to significant increases in RNA abundance of this region subsequent to TCIE. Unlike the consistent decrease in LAT transcription following iontophoresis, the RNA abundance of the ICP4 region is transient in nature, leading us to further speculate that LAT remodeling and the subsequent decreased LAT transcription facilitate the transient increase in lytic transcript abundance, and that chromatin remodeling of the LAT may ultimately be responsible for events leading to *in vivo* reactivation.

Previous studies identified changes in the acetyl-H3K9K14 associated with the LAT and IE regions of HSV-1 following the application of a reactivation stimulus in mice [19], [59]. However, previous reports failed to connect epigenetic changes to the latent viral genome with increased lytic transcription, raising the question as to the relevance of epigenetic changes in reactivation. Here, we show a 2-6-fold increase in ICP4 transcript abundance is preceded by a significant (but transient) increase in the euchromatic histone mark H3K4me2 following the application of a reactivation stressor in the efficient HSV-1 reactivating strain, McKrae. This increase is paralleled to decreased enrichment and subsequent transcript abundance of the LAT region of the genome. In contrast, we could not detect any significant changes in either the enrichment of H3K4me2 of the LAT and IE regions of the inefficient reactivating strain, KOS following TCIE through 4 hours. This finding, coupled with the unchanging abundance of LAT RNA indicate that changes in histone markers on the LAT and the IE regions of HSV-1 may be a crucial factor in *in vivo* reactivation. To our knowledge, this is the first study to link changes in euchromatic marks of LAT and the IE regions exclusively to a high phenotypic reactivating strain of HSV-1.
